# Seasonal Influence on the Performance of Low-Cost NO_2_ Sensor Calibrations

**DOI:** 10.3390/s21237919

**Published:** 2021-11-27

**Authors:** Sjoerd van Ratingen, Jan Vonk, Christa Blokhuis, Joost Wesseling, Erik Tielemans, Ernie Weijers

**Affiliations:** 1 National Institute for Public Health and the Environment (RIVM), P.O. Box 1, 3720 BA Bilthoven, The Netherlands; jan.vonk@wur.nl (J.V.); christa.blokhuis@wur.nl (C.B.); joost.wesseling@rivm.nl (J.W.); erik.tielemans@rivm.nl (E.T.); ernie.weijers@rivm.nl (E.W.); 2 Wageningen Livestock Research, P.O. Box 338, 6700 AH Wageningen, The Netherlands; 3 Consumption & Healthy Lifestyles, Wageningen University & Research, P.O. Box 8130, 6700 EW Wageningen, The Netherlands

**Keywords:** NO_2_ sensor, ozone, calibration, validation, measurement uncertainty, multivariate linear regression, seasonal influence

## Abstract

Low-cost sensor technology has been available for several years and has the potential to complement official monitoring networks. The current generation of nitrogen dioxide (NO_2_) sensors suffers from various technical problems. This study explores the added value of calibration models based on (multiple) linear regression including cross terms on the performance of an electrochemical NO_2_ sensor, the B43F manufactured by Alphasense. Sensor data were collected in duplicate at four reference sites in the Netherlands over a period of one year. It is shown that a calibration, using O_3_ and temperature in addition to a reference NO_2_ measurement, improves the prediction in terms of R^2^ from less than 0.5 to 0.69–0.84. The uncertainty of the calibrated sensors meets the Data Quality Objective for indicative methods specified by the EU directive in some cases and it was verified that the sensor signal itself remains an important predictor in the multilinear regressions. In practice, these sensors are likely to be calibrated over a period (much) shorter than one year. This study shows the dependence of the quality of the calibrated signal on the choice of these short (monthly) calibration and validation periods. This information will be valuable for determining short-period calibration strategies.

## 1. Introduction

Within the framework of the European Air Quality Directive [1], it is possible to use supplementary techniques for indicative measurements. Emerging low-cost sensor technology may fulfill this role. Compared to reference instruments, the use of air quality sensors in monitoring would reduce costs and allow for high-resolution spatial–temporal coverage [2,3,4]. However, multiple studies testing the capabilities of air quality sensors under field conditions indicate highly variable behavior when comparing the sensor-reference data outputs [5,6]. Sensors react to interfering pollutants or display unrealistic concentrations at high temperature or relative humidity [7,8]. To overcome this challenging behavior, calibration algorithms (models) need to be more refined in order to produce reliable data [9]. Then, after subsequent validation, potential applications of a single or network of low-cost sensors for air quality monitoring as well as citizen science can be judged to their full extent [10,11,12,13,14].

The calibration of low-cost sensors can be explored in various ways. Using default relations provided by the manufacturer sometimes results in non-physical negative concentrations and large differences between sensor and reference data. Using multilinear regression models and including the temperature and relative humidity in ambient air improves the results, indicating that calibration in the field is highly recommended. Spinelle et al. [15] showed that O_3_ sensors behave more reliably when using single regression while NO_2_ sensors are preferably calibrated by employing supervised learning techniques, indicating that different approaches might be necessary depending on the type of sensor. Hasenfratz et al. [16] compared backward and instant calibration to traditional forward calibration and showed that such an approach improves data accuracy considerably.

In our study, the behavior of individual sensors is discussed by designing several (multi-)linear regression models. These were constructed for the Alphasense NO2-B43F sensor [17] to predict continuous reference data and are based on the predictor variables: NO_2_ measured by the sensor, temperature, relative humidity and ozone (either by reference instrumentation or by a sensor) in ambient air. Our analyses also include interaction (or cross) terms (the product of two or more independent predictor variables). Such terms describe the effect that the relationship between a given (independent) predictor and the outcome may also depend on other predictor variables. The performance of such models is expressed here in several validation metrics including the expanded relative measurement uncertainty which, following [18,19], was compared with the Data Quality Objectives (DQO) as defined in the Air Quality Directive.

As far as we know, most research so far has focused on experimental periods shorter than one year. The data collection in this study covers one year. This allowed for a detailed assessment of the effect of specific choices for calibration and validation periods on the sensor performance in detail here. Dividing the dataset into months, it is shown that the choice of calibration/validation period can affect the performance of a calibrated sensor considerably. We show, e.g., that in terms of R^2^, the summer calibration works reasonably well in winter, but winter calibration does not perform well in summer. Using May 2018 as a calibration dataset to (backwards) predict all other months gives the best results throughout the year, which can be explained by the optimal ranges for the predictor values during May 2018.

## 2. Experimental Setup

### 2.1. Low-Cost Sensors

The focus of our analysis is on nitrogen dioxide (NO_2_); ozone is measured by either a reference instrument or sensor for calibration purposes only. Here, we chose the Alphasense NO2-B43F, a popular, low-cost electrochemical sensor for measuring ambient NO_2_. This sensor is part of the platform developed by the Joint Research Centre (JRC) of the European Commission: AirSensEUR. It is an air quality monitoring system developed as an open software/open hardware object and complies with the INSPIRE Directive [20]. Apart from the Alphasense NO_2_ sensor, the AirSensEUR configuration used in this experiment also contained a Membrapor O_3_/M-5 for ozone measurements and dedicated meteorological sensors for temperature, air pressure, and relative humidity. In the AirSensEUR, the T/P/RH board and sensor surfaces are located outside the box directly in the ambient air.

Measurement samples are sent to a host board, which supplements the measurements with geographical coordinates and then sends all data to a database [21]. In the case of platform dropout, a manual re-start was performed. The calibration given by the manufacturer was not used in our study. Sensor measurements are provided as counts from the A/D converter (corresponding to the 16-bit AD-conversion applied in the AirSensEUR shield). Currents from the sensors in milliamperes (mA) are converted to digital values according to the configuration parameters given by the manufacturer. Sensor readings of temperature, air pressure and relative humidity are converted according to the indication of the manufacturer to degrees Celsius, millibar and percentages, respectively. The raw measurement data provided by the sensors are stored in a central database on a 1 min base.

### 2.2. Field Deployment

**Figure 1:**
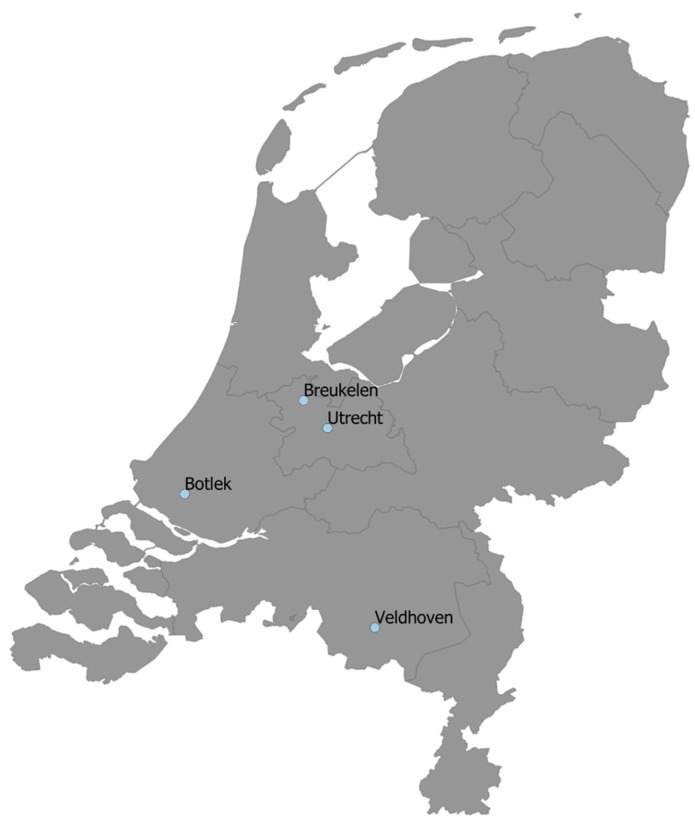
Locations of reference sites in the Netherlands used in this study.

For a period of one year (June 2017 until May 2018), eight AirSensEUR platforms were deployed at four reference sites being part of the of the National Air Quality Monitoring Network (LML) in the Netherlands (two sensors per site). The types of sites were urban background (Veldhoven), street (Utrecht), motorway (Breukelen) and industry (Botlek) (see Figure 1). Every reference site is equipped with reference gas analyzers for NO_2_ (chemiluminescence; Teledyne API 200E except Botlek, Thermo 42i) and O_3_ (UV photometry; Thermo 49i except Botlek, Thermo 49C) (Nguyen, 2009). The reference analyzers are calibrated in the field using filtered zero air and span value.

### 2.3. Data Filtering

Due to non- or malfunctioning of the sensor or hardware (sensor systems being prototypes), there were episodes in the time series with missing or distorted data. These were visually removed from the dataset. Additional filtering was done to remove suspected outliers: minute-based measurements lying outside ±10 times the standard deviation range of the annual average level are discarded. This criterion was used for its simplicity and effectiveness. The resulting time coverage per sensor of data available for further processing is given below in Table 1 for each station. The filtering process resulted in an average data coverage over the year of 68%. From the one-minute sensor, hourly values were derived to enable a direct comparison with the hourly reference data and to improve the signal-to-noise ratio.

## 3. Calibration and Validation

### 3.1. Calibration

Within this study, a (multivariate) regression approach will be used to calibrate the raw signal of the sensors. The calibration will be performed using hourly averaged predictors. As meteorology and the presence of ozone in ambient air both affect the response of low-cost sensors, the following predictors for the NO_2_ signal of the reference measurements will additionally be considered:
•Sensor NO_2_ signal:sensorNO_2_;•Reference O_3_ concentration:refO_3_;•Sensor O_3_ signal:sensorO_3_;•Sensor temperature signal: sensorT;•Relative humidity at the nearest weather station:RH.

Using these predictors, eight multivariate regression variants are examined:

1. refNO_2_~sensorNO_2_;
2. refNO_2_~sensorNO_2_, sensorT;
3. refNO_2_~sensorNO_2_, sensorO_3_;
4. refNO_2_~sensorNO_2_, refO_3_;
5. refNO_2_~sensorNO_2_, sensorT, sensorO_3_;
6. refNO_2_~sensorNO_2_, sensorT, refO_3_;
7. refNO_2_~sensorNO_2_, sensorT, sensorO_3_, RH;
8. refNO_2_~sensorNO_2_, sensorT, refO_3_, RH.

Each variant is fitted using the sensor datasets collected at the reference sites. When the regression uses two or more predictors, this regression will also contain cross (or interaction) terms of these predictors. For example, “refNO_2_~sensorNO_2_, sensorT” refers to a multivariable regression fit that also includes the product of sensorNO_2_ and sensorT and the regression “refNO_2_~sensorNO_2_, sensorT, sensorO_3_, RH” contains a product of sensorNO_2_, sensorT, sensorO_3_, and RH.

Terms in the regression containing only one predictor are known as the ‘additive’ model and investigate only the main effects of the predictors (where it is assumed that the relationship between a predictor variable and the outcome is independent of other predictors). The incorporation of a product of two or more (independent) predictors in a regression is motivated by the occurrence of an ‘interaction effect’. These interactions occur when the effect of an independent predictor variable on a dependent variable changes, depending on the value(s) of one or more other predictor variables. The understanding of the physical significance of cross terms is quite challenging. Some cross terms seem to improve the performance of the calibration model, while for others the improvement seems negligible. Within this study, it was decided to include these cross products in the calibration in order to make use of the possible added value. Additional study of the relevance of individual cross terms is not presented in this article.

### 3.2. Validation

To evaluate the sensor performance after applying the different calibration models, the calibrated sensor data were then validated with NO_2_ data from the reference equipment by orthogonal regression. As validation metrics, R^2^ (coefficient of determination), slope and intercept, prediction error (RMSE) and the measurement uncertainty were calculated. The measurement uncertainty is compared to the Data Quality Objective (DQO) for indicative methods that corresponds to a relative expanded uncertainty of 25% for NO_2_ at the limit value set by the European Directive [1]. The estimation of the uncertainty, which corresponds to the relative expanded uncertainty *Ur*, is carried out following Equation (1) using the slope and intercept of the orthogonal regression equation and the sum of the square of the residuals:

(1) $$Ur\left(Yi\right)=\frac{2\sqrt{\frac{1}{n-2}\sum_{i}{\left(Y_{i}-\left(intercept+slopeX_{i}\right)\right)}^{2}-u^{2}\left(X_{i}\right)+{\left(intercept+\left(slope-1\right)X_{i}\right)}^{2}}}{Yi}$$

with *u*(*X_i_*) = 1.8% × *X_i_* being the between-sampler uncertainty of the reference equipment. Details of the calculation of the orthogonal regression can be found in the Guide for the Demonstration of Equivalence [22].

## 4. Results and Discussion

### 4.1. Presentation of the Dataset

**Figure 2:**
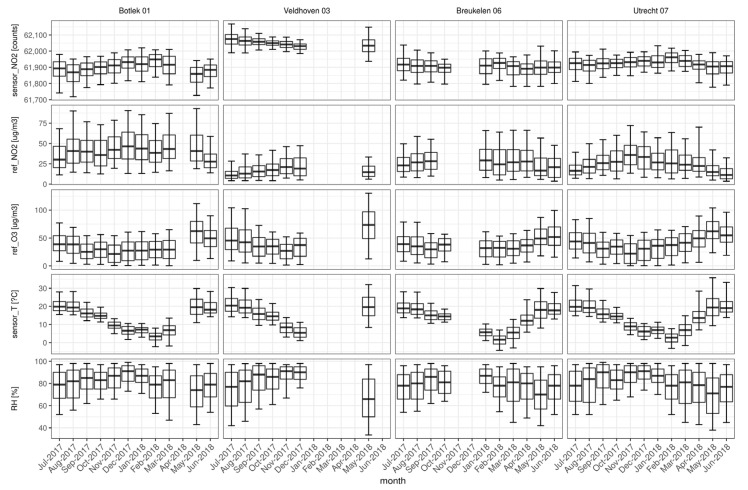
Box-and-whisker plots of reference measurements and predictors used in the calibration of the sensors. The horizontal line indicates the median. The boxes and whiskers show, respectively 25% to 75%, and 5% to 95% of the data.

To show the variability over location and time of the predictors used in the calibrations, box plots are presented on a monthly basis (Figure 2). The lines extending vertically from the boxes indicate the variability outside the upper and lower quartiles (denoted by 5th and 95th percentiles). The variables included are the raw sensor NO_2_ data (counts), reference measurement of NO_2_ (refNO_2_ in µg/m^3^) and O_3_ (refO_3_ in µg/m^3^), temperature (sensorT in °C) and relative humidity (RH in %) measured at the nearest weather station. Only months with at least 200 h (8 days) of measurement data are included (leading to the loss of monthly statistics at Veldhoven and Breukelen).

The time series of sensorT and RH indicate that the meteorological behavior at the four sites is quite similar to the monthly (median) temperatures ranging between 0 and 20 °C and 65–95% RH. This was anticipated in a dominant maritime climate (mild winters, cool summers) and the limited distances (<100 km) between measurement sites. The annual cycles of sensorT and RH show maximum temperatures in the period May–August and a minimum relative humidity in May at all four sites. 

Still recognizable is the annual variation of NO_2_ and O_3_ (measured with reference instruments). As expected, both pollutants behave oppositely throughout the year. This is most apparent at the Utrecht site where the lowest NO_2_ (and highest O_3_) median levels occur in May and June (coinciding with high temperatures). On average, the highest NO_2_ concentrations occur at the industrial site Botlek, probably due to the local emissions from heavy industry and transport. Figure 2 shows that concentrations of NO_2_ are lowest at the urban background site Veldhoven (with relatively high levels of ozone), whereas the traffic-dominated sites Breukelen and Utrecht show intermediate values. For ozone, the annual behavior appears comparable at the four sites.

### 4.2. Linear Regression (LR)

**Figure 3:**
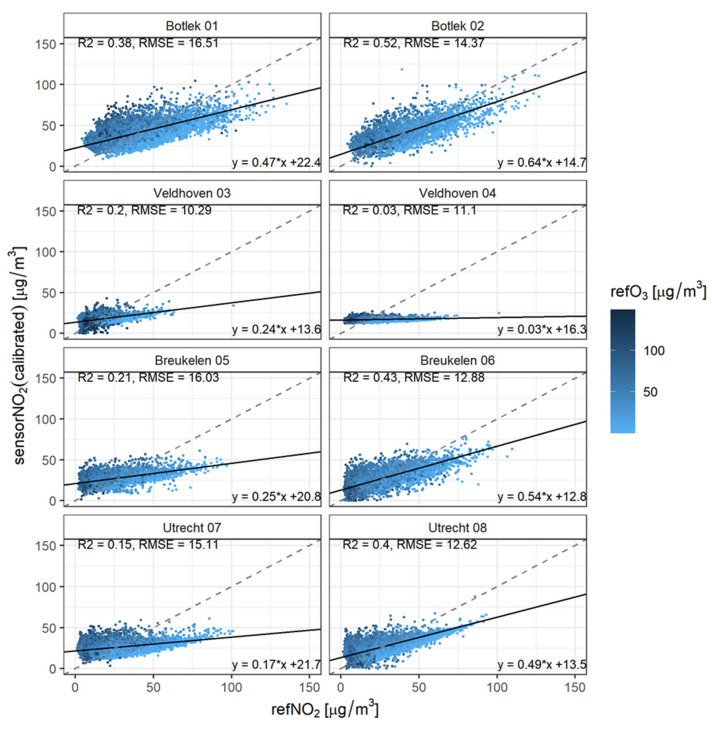
Scatter plots for concentration prediction by single linear calibration. The dot colors correspond to ambient O_3_ concentration. The dashed line represents Y = X; the solid line follows from the orthogonal fitting between the calibrated sensor and reference concentration data.

Single linear regression is used to calibrate the sensor data using all available sensor and reference measurements. Subsequently, a univariate orthogonal regression has been used to determine the slope and intercept of the calibrated sensors with respect to the reference measurements. In this case, the validation dataset equals the calibration data. Validation metrics are given per sensor in Figure 3 (and summarized in Table 2). The color of the data points indicates whether ozone concentrations are high or low (darker means higher). The dashed line represents Y = X and the solid line follows from the orthogonal fitting between the calibrated sensor and reference concentration data.

The performance of a prediction, based on a single linear regression between the reference and sensor measurements, proves to be of poor quality. The relative spread between the sensor and reference measurements seems to decrease with increasing NO_2_ and decreasing O_3_. This could be explained by the measurement error of the NO_2_ sensor itself and cross-sensitivity of the NO_2_ sensor to O_3_. A univariate calibration model obviously cannot correct for the interference by ozone. High NO_2_ and low ozone levels predominantly occur during wintertime. This is also the case when the prediction based on single linear regression performs best. We will discuss this in more detail below.

To check for possible time-linear drift of the calibrated sensors, the difference between the monthly averages of the calibrated sensors and the reference equipment is given in Figure S1 of the supplementary materials. Because of the large fluctuation of this difference throughout the year, it is hard to discern a linear trend, indicative of such a drift.

### 4.3. Multivariate Linear Regression (MLR)

#### 4.3.1. Performance Metrics

**Figure 4:**
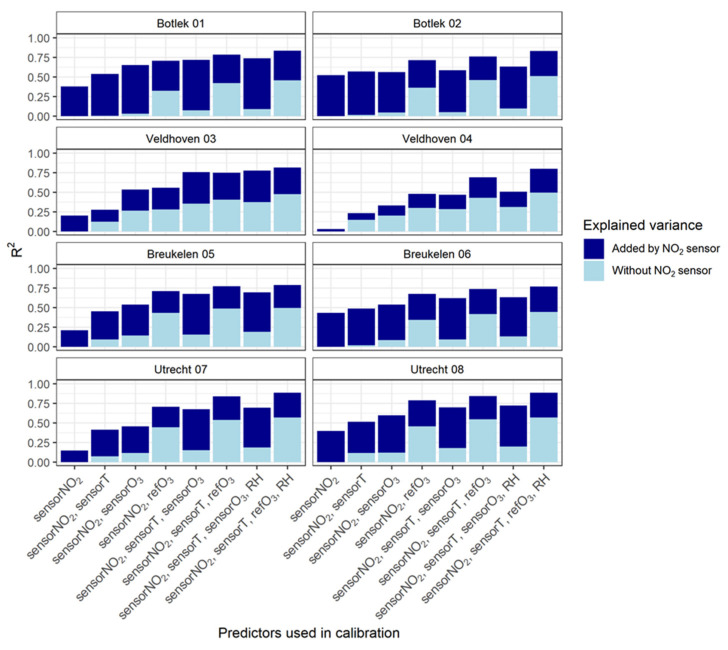
R^2^ (coefficient of determination) for the reference NO_2_ concentrations versus eight calibration models (horizontal axis). The light blue part in each bar shows the calculated R^2^ of the calibration model with the NO_2_ sensor data excluded. The dark blue part represents the variance explained by the NO_2_ sensor.

After applying the various multivariate regression models for the calibration, coefficients of determination (R^2^) were calculated to estimate the variance in the dependent variable (refNO_2_) that is predictable from the (independent) variables (predictors). The results are shown in Figure 4. As our focus is on the behavior of the NO_2_ sensor, it is of interest to estimate to what extent the outcome of the regressions is determined by the sensor itself. Therefore, the vertical bars are divided into two parts to distinguish between the variances explained by the sensor predictor (dark blue) and by the remaining predictors (light blue). The light blue part of the bar thus shows the performance of the regression model without actually making use of NO_2_ sensor data.

As expected, MLR models perform better than the LR model (given by the first bar on the left in Figure 4: model 1). Including the temperature in the model as a predictor variable improves R^2^ (model 2). The improvement (compared to model 1) is even larger when ozone data (either measured by the O_3_ sensor or derived from the reference instrument: models 3 and 4) is added to the calibration confirming (again) that part of the explained variance is due to the cross-sensitivity of the NO_2_ sensor to O_3_. The inclusion of reference O_3_ data instead of sensor O_3_ data always leads to a better agreement. This is (partly) because the ambient O_3_ concentrations anti-correlate relatively strongly with ambient NO_2_ concentrations (Figure 2).

Adding the sensor temperature variable (models 5 and 6) improves the results even more. The incorporation of the relative humidity parameter (seventh and eight bar) only produces a minor improvement (if any). Apparently, the use of the temperature variable within the models accounts sufficiently for the explained variance, which can be understood from the similar but opposite temporal behavior of these meteorological variables.

The difference between monthly averages of the sensors, calibrated using model 6 and the reference equipment is given in Figure S2 of the supplementary materials. The figure does not indicate a time-linear drift between calibrated sensors and reference equipment.

**Figure 5:**
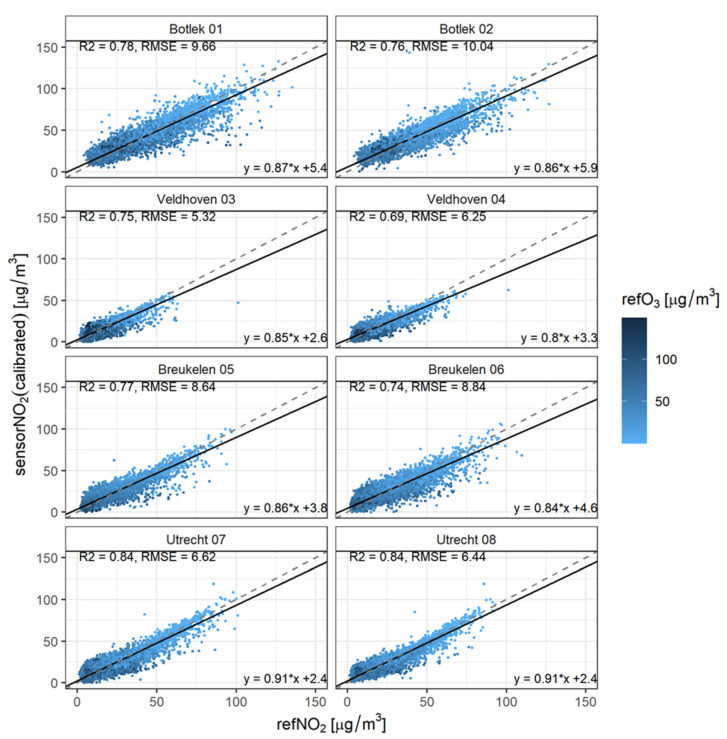
Like Figure 3 but this time for calibration: refNO_2_~sensorNO_2_, sensorT, refO_3_. The colors correspond to the ambient O_3_ concentration.

Figure 4 shows that the inclusion of NO_2_ sensor data in the calibration models indeed improves the predictive quality. Even when a large part of the variability can be explained by the correlation of NO_2_ with O_3_ alone, the NO_2_ sensor is still able to establish a significant increase in R^2^. One of the best performances is observed when the MLR regression incorporates reference O_3_, sensor temperature and sensor NO_2_ data (model 6). This is demonstrated in more detail in Figure 5 where (like in Figure 3) orthogonal regression is used to validate the performance of the calibration model. The coloring of the data points indicates the level of the ozone concentrations.

Compared to Figure 3 (single linear regression approach), the calibration performance improves considerably in terms of R^2^, RMSE, slope and intercept. Additionally, note in Figure 5 that the spread in the dataset is reduced. The improvement from LR to MLR with predictor variables sensorNO_2_, sensorT and refO_3_ data is summarized in Table 2. A similar figure, but now for a calibration using sensorNO_2_, sensorT and sensorO_3_ has been added to the supplementary materials (Figure S3).

#### 4.3.2. Relative Measurement Uncertainty

**Figure 6:**
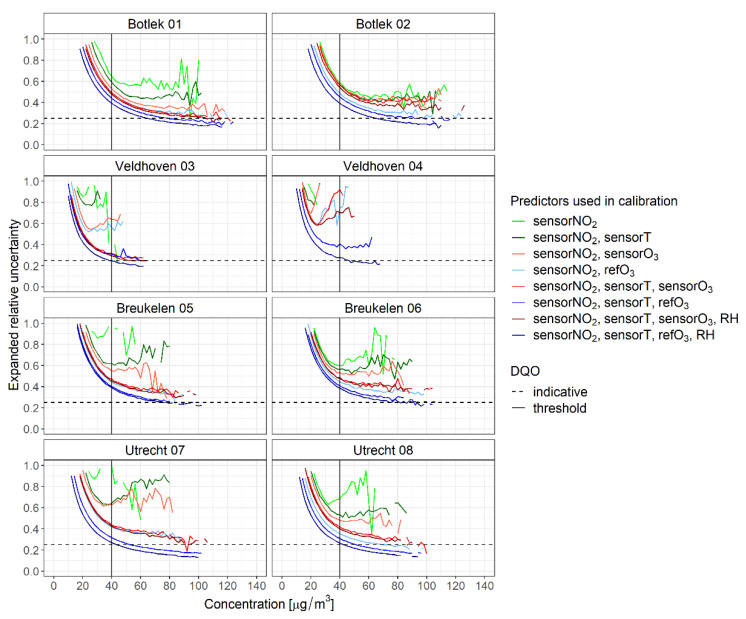
Relative expanded uncertainty of the predicted values versus reference data as a function of the level of NO_2_ for the calibration models. Green, red and blue lines, respectively indicate calibrations without ozone, calibrations with sensor ozone and calibrations with reference ozone. The dashed line shows the DQO for indicative measurements.

In addition to the abovementioned performance metrics, corresponding measurement uncertainties estimated using Equation (1) are compared with the Data Quality Objectives (DQO) for indicative measurements (i.e., 25% for the 95% confidence level at the limit value of 40 µg/m^3^). The measurement uncertainties were calculated using the full dataset. The result is given in Figure 6 for every model as a function of the level of the NO_2_ concentration (as measured at the reference stations).

For most models, the DQO for indicative measurements is not met. Clearly, calibration models including an ozone predictor (sensor or reference) perform significantly better, especially at higher concentrations. As might be anticipated from the previous results, the calibrations using all available predictors (based on models 7 and 8 with ozone either from the sensor or from the reference) yield the lowest relative expanded measurement uncertainty at every measurement site. For these models, the uncertainties estimated at some stations appear very close to, or even comply with the DQO for indicative measurements.

Since each measurement location is equipped with two sensor units, a comparison between sensor data provides an indication of the sensor-to-sensor variability. For the (uncalibrated) NO_2_ sensor signals such a comparison is presented in Figure S4 of the supplementary materials. Although beyond the scope of the work presented here, this could be used to break down the estimated uncertainty of the calibrated sensors into components associated with, e.g., the sensor-to-sensor variability and the calibration uncertainty.

#### 4.3.3. Calibration and Validation by Monthly Datasets

**Figure 7:**
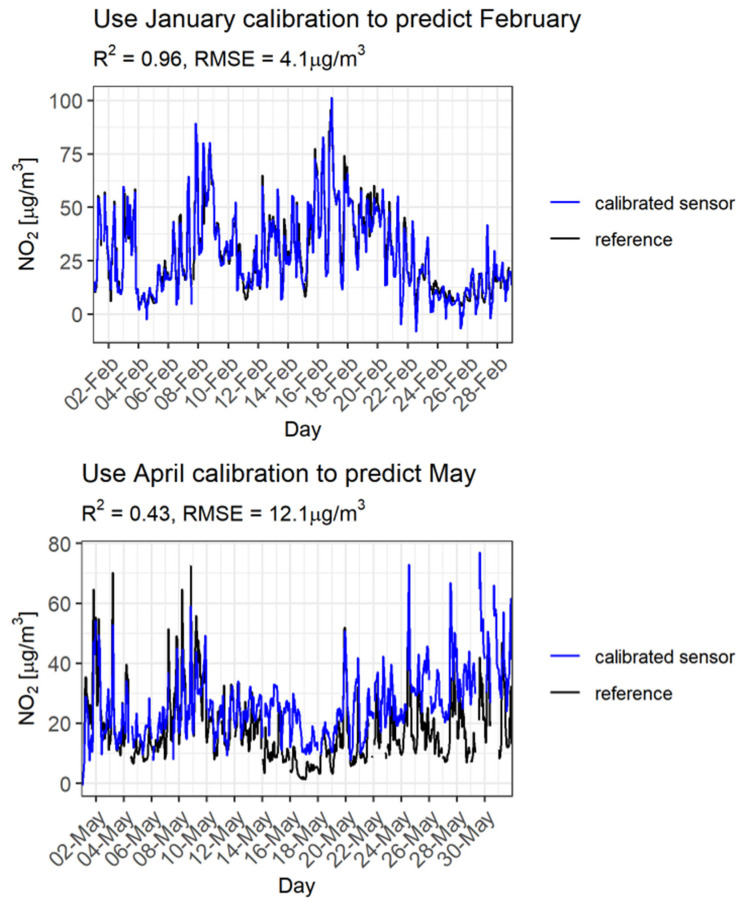
Predictions for the calibration “refNO_2_~sensorNO_2_, sensorT, refO_3_”. Black line: reference. Blue line: prediction. Top: Prediction for February based on a calibration in January. Bottom: Prediction for May based on a calibration in April.

So far, the testing of the sensor models’ performances has been restricted to the one-year datasets with calibration and validation periods overlapping in time. Although not systematically investigated, the lifetime of an electrochemical sensor is reported to be 1–2 years. Therefore, calibrations should be conducted at shorter time intervals, e.g., one month (also for practical reasons). In addition, it is of interest to validate the calibrated sensors for shorter time spans to investigate whether they perform better or worse in different validation periods (e.g., the entire measurement period of one year or a specific month). To study this, model 6 is applied to monthly subsets of the measurement data (each consisting of at least 200 hourly values). To visualize how this works out, Figure 7 shows predictions based on two different calibration/validation month combinations compared with reference concentrations. In this example, the data from the Utrecht measurement site and sensor 07 have been used.

The top part of Figure 7 shows that a model ‘trained’ with data from January can make good predictions for February. Trained with data from April, the model overpredicts the measured concentrations in May from the second half of the month (bottom part of Figure 7). This could be explained by different reference concentrations and meteorological circumstances from those encountered in the calibration month. 

**Figure 8:**
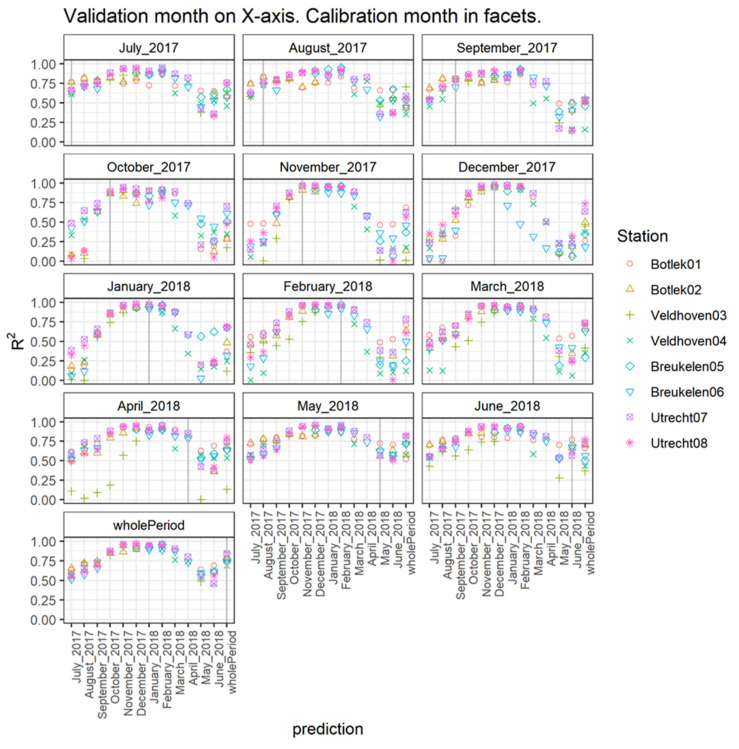
R^2^ for model 6 for each combination of calibration and validation period (month or entire year). The month used for calibration is given in the title of the subgraphs. The validation month is given on the *X*-axis.

The combined results of this approach for all stations and available months are presented in Figure 8 where performances are expressed in terms of explained variance (R^2^). The title in each subgraph corresponds to the month providing the calibration data. The *X*-axis corresponds to the (monthly) period for which the validation is done, i.e., the top-left graph shows the R^2^ between the NO_2_ of the sensors and that of the co-located reference measurements for the months July 2017–June 2018, when the sensors were calibrated using the data from July 2017. The vertical gray line indicates when the calibration and validation month coincide. Results to the right of this line are based on a calibration that was determined before the validation was performed. Results to the left of the line indicate how the calibrated sensor predicts the concentrations per month when it is calibrated using the dataset from a future month. The last tick mark on the *X*-axis gives the R^2^ when the entire year is used as validation data (discussed in previous paragraphs).

In general, it can be concluded that the validation period itself is the most important factor determining the quality of the calibration based on MLR (model 6). Irrespective of the calibration month, the period November until February systematically shows the highest explained variances. When calibration is carried out in a winter month, the validation shows the most accurate results for the winter period but is accompanied by a relatively (very) low performance in the summer. Using a calibration based on the period May–August, the performance in winter remains accurate while acceptable (R^2^ > 0.5) performance is observed for the remaining validation months. The calibration using the entire measurement dataset (last subgraph) also performs best in wintertime. More specifically, when studying the results per month, calibrating the sensor in May yields the best result for every month of the year and is rather similar to the results of a calibration based on the entire year. This could be explained by the predictor variables all having a high variability during this month. Due to annual meteorological variability, this may be different for other years and will obviously change in other climate zones.

**Figure 9:**
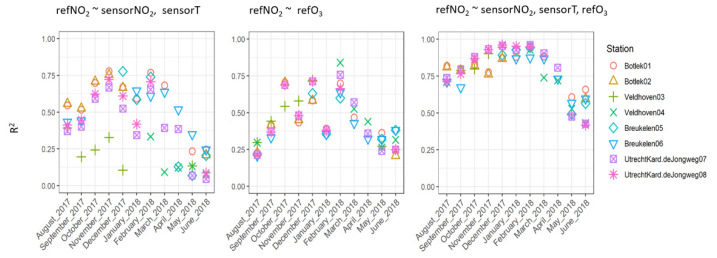
R^2^ for calibration based on three different regressions. All available data in the months prior to the validation month have been used to derive the calibration.

Comparing Figure 2 and Figure 8, it is noted that the quality of the prediction in terms of R^2^ corresponds with the average levels of the NO_2_ concentrations as well as the average ozone concentrations and sensor temperatures. Months with high sensorNO_2_ levels in combination with low ozone levels and sensor temperature generally yield the highest values of R^2^. Because these predictors are highly intercorrelated, the variance explained by the individual predictors is given below (Figure 9) in the left and middle subgraphs, equaling around 0.75 at the highest. In this case, the prediction model that is validated with the month on the *X*-axis is based on a calibration using data from all months preceding this validation month. The explained variance of the combined predictors is shown in the right graph, revealing considerably larger values for R^2^ (up to 0.95). Just as was already demonstrated using the full (one year) dataset in Figure 4, the sensorNO_2_ signal has an important role in explaining reference NO_2_ levels on a monthly basis.

## 5. Discussion and Conclusions

In this study the performance of low-cost sensors, calibrated using (multiple) linear regression, is investigated in two ways. First, by varying the set of predictor variables used in the calibration while allowing for the products of predictor variables. Second, by varying calibration and validation month, given that the full extent of the dataset covers one year. 

The performance of a prediction, based on linear regression between reference and sensor measurements, proves to be of poor quality; the coefficients of determination (R^2^) are less than or equal to 0.54. To improve these predictions, a MLR modelling approach using predictors like temperature, relative humidity and ozone in ambient air is examined. Possible interaction effects are approximated by adding the product of predictor variables to the calibration equations. For these kinds of calibration models, R^2^ increases to 0.69–0.84, substantially higher than with the linear regression approach.

The best performing calibrations always include an ozone predictor (either from reference measurements or sensor measurements), which accounts for the part of the explained variance that is due to the cross-sensitivity of the NO_2_ sensor to ambient O_3_. The use of reference O_3_ data instead of sensor O_3_ data in the calibration improves the performance, which is most likely due to the cross-sensitivity of the ozone sensor for NO_2_. Adding the temperature to the calibration equation improves R^2^ even further. The incorporation of the relative humidity parameter in the calibration only results in a minor improvement (which is probably caused by the strong anti-correlation with temperature). We therefore conclude that ambient ozone concentrations and temperature must be taken into account in the calibration of the low-cost NO_2_ sensors discussed here.

Compliance with the Data Quality Objective for indicative methods (95% CI uncertainty of 25% for yearly average NO_2_ at the limit value (40 ug/m^3^) set by the European Directive) is also investigated using data from the full (one year) measurement period. The uncertainties estimated at some stations (urban background or in a street) turn out to be very close to or in compliance with the Data Quality Objective.

The testing of the sensor calibration usually involves calibration and validation periods shorter than one year. We show that the choice of calibration/validation period can affect the performance of a sensor considerably. When sensors are calibrated in a winter month (using the best performing calibrations in this study), optimal results are obtained for the remaining winter months, but the summer period shows a (very) low performance in this case. This might be due to the low ozone concentrations during wintertime. When using a calibration based in summer, performance in terms of R^2^ in winter remains quite good and performances for the remaining months remain acceptable. Possibly valuable for practical use is the observation that for the specific meteorological conditions and ambient NO_2_ and O_3_ concentrations in the dataset, a sensor that is calibrated in May yields the most accurate results for the remaining 11 months and is, in addition, rather similar to the results of a calibration based on the entire year. One possible explanation is that during the month of May the important predictor variables (NO_2_, O_3_, T, RH) show the large variations needed for an adequate calibration. Conditions in the month of May 2018 seem sufficiently representative for both winter and summer variability in atmospheric behavior.

It is worth noting that the O_3_ reference measurements that were input into the optimal calibrations in this paper will, in practice, not be available at the location of the low-cost sensors. In future, it should therefore be investigated to what extent using a nearby O_3_ station or an interpolation map of high-quality O_3_ information in the calibration algorithms influences the quality of the calibration (compared to the use of a local O_3_ sensor).

## Figures and Tables

**Table 1:** Data coverage at the measurement sites for each sensor system. Number of hours with valid hourly averaged sensor output as a percentage of the total number of hours in the measurement period (one year).

Station/Sensor No.	%
Botlek 01	91
Botlek 02	55
Veldhoven 03	59
Veldhoven 04	54
Breukelen 05	38
Breukelen 06	78
Utrecht 07	100
Utrecht 08	71
Average	68

**Table 2:** Calibration performance expressed in R^2^, RMSE, slope and intercept. Numbers in parentheses are the results from single linear regression.

MLR (LR)	Sensor	Slope	Intercept	R^2^	RMSE
Botlek	01	0.87	5.41	0.78	9.66
		(0.47)	(22.39)	(0.38)	(16.51)
	02	0.86	5.89	0.76	10.04
		(0.64)	(14.73)	(0.52)	(14.37)
Veldhoven	03	0.85	2.57	0.75	5.32
		(0.24)	(13.63)	(0.2)	(10.29)
	04	0.8	3.32	0.69	6.25
		(0.03)	(16.34)	(0.03)	(11.1)
Breukelen	05	0.86	3.78	0.77	8.64
		(0.25)	(20.83)	(0.21)	(16.03)
	06	0.84	4.55	0.74	8.84
		(0.54)	(12.81)	(0.43)	(12.88)
Utrecht	07	0.91	2.39	0.84	6.62
		(0.17)	(21.71)	(0.15)	(15.11)
	08	0.91	2.36	0.84	6.44
		(0.49)	(13.48)	(0.4)	(12.62)

## Data Availability

Please contact sjoerd.van.ratingen@rivm.nl for the sensor and reference measurements used in this study.

## Supplementary Materials

The following are available online at https://www.mdpi.com/article/10.3390/s21237919/s1, Figure S1: Monthly averaged difference between the NO_2_ sensor, calibrated using simple linear regression and the reference NO_2_ concentration. Figure S2: Monthly averaged difference between the NO_2_ sensor, calibrated using multivariate linear regression (based on the sensor NO_2_ signal, the sensor temperature and the reference O_3_ concentration) and the reference NO_2_ concentration. Figure S3: Scatterplots for sensors, calibrated with the NO_2_ sensor signal, the sensor temperature and the O_3_ sensor signal. Figure S4: Scatter of NO_2_ sensor signal of co-located sensor pairs at the four measurement locations.
