# Supplementary material for: Seasonal Influence on the Performance of Low-Cost NO2 Sensor Calibrations
Source: Sensors (Basel). 2021 Nov 27;21(23):7919. doi: 10.3390/s21237919 (PMC8659619; doi:10.3390/s21237919)
Supplement: Supplementary file 1 [file sensors-21-07919-s001.zip › sensors-1442378-supplementary.pdf]

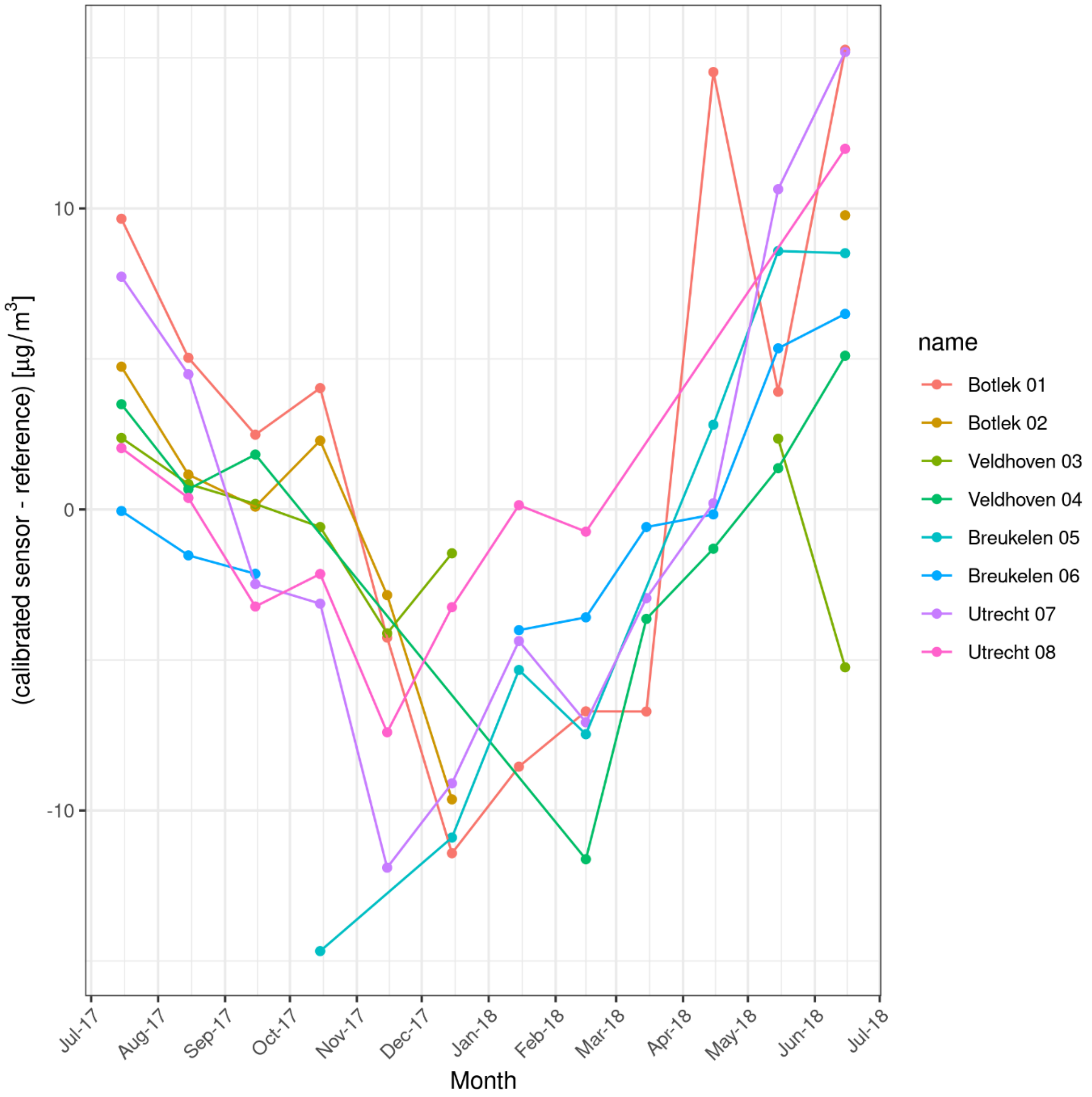

Figure S1. Monthly averaged difference between the NO_2 sensor, calibrated using simple linear regression and the reference NO_2 concentration.

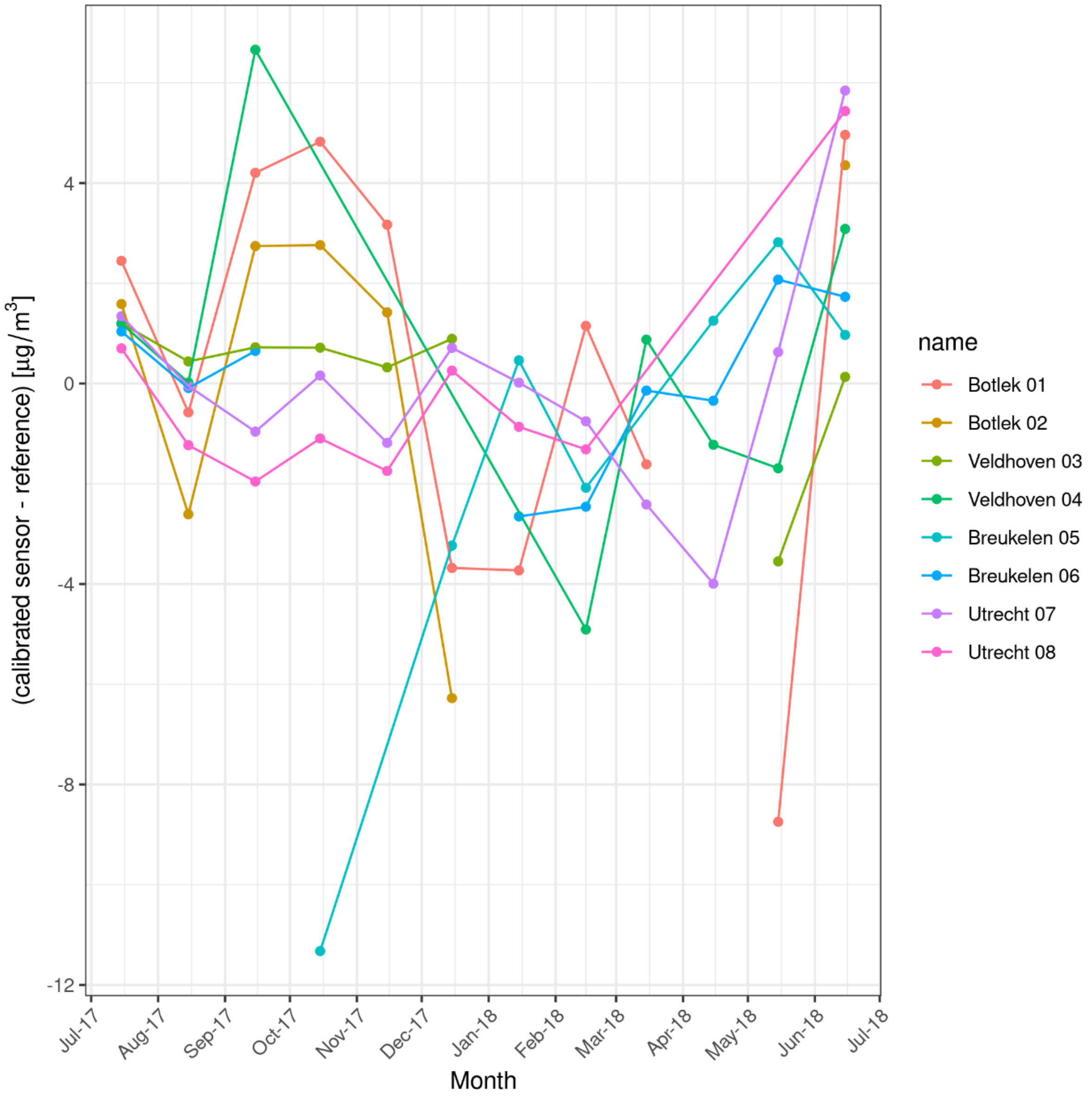

Figure S2. Monthly averaged difference between the NO_2 sensor, calibrated using multivariate linear regression (based on the sensor NO_2 signal, the sensor temperature and the reference O_3 concentration) and the reference NO_2 concentration.

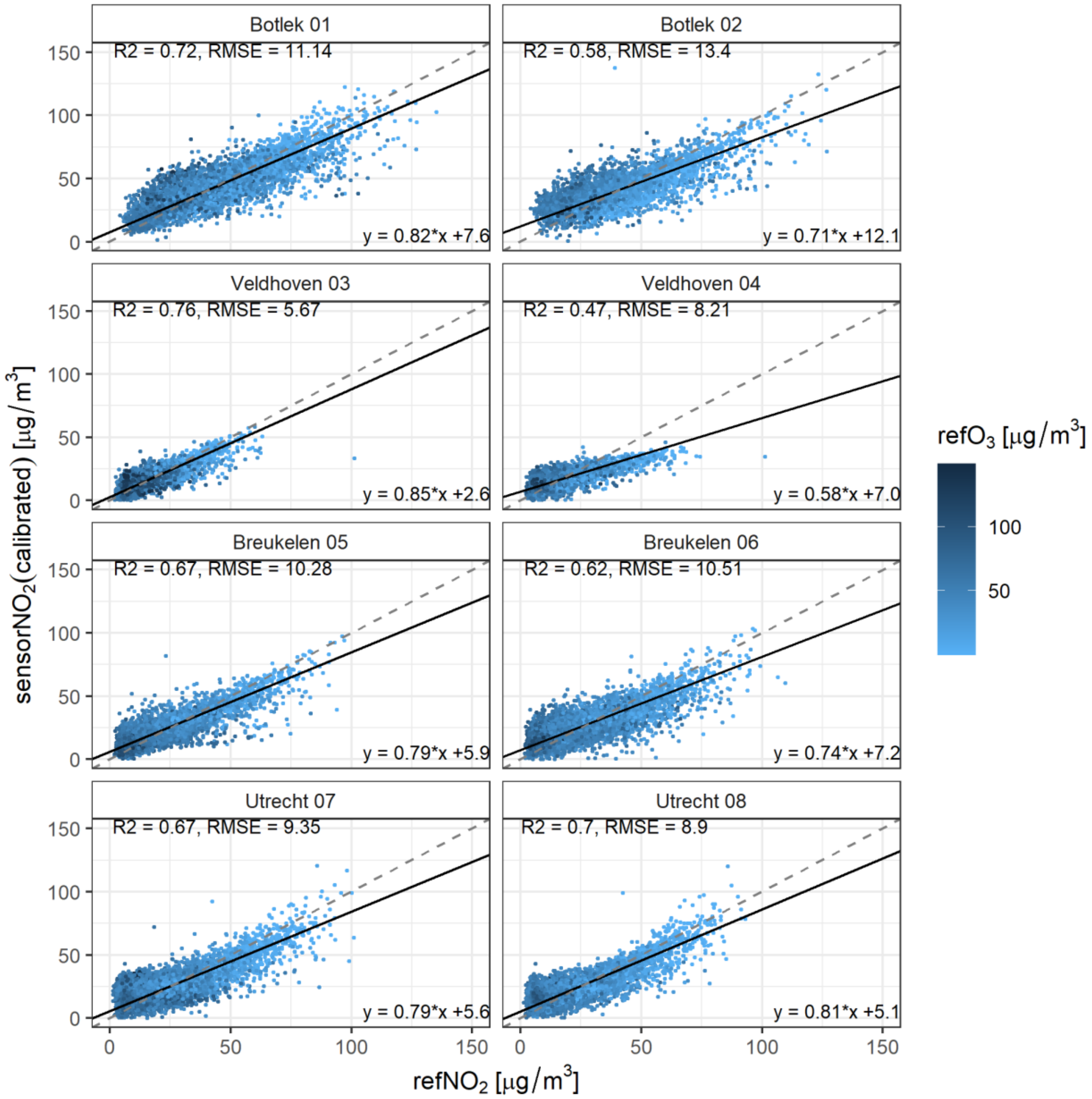

Figure S3. Scatterplots for sensors, calibrated with the NO_2 sensor signal, the sensor temperature and the O_3 sensor signal.

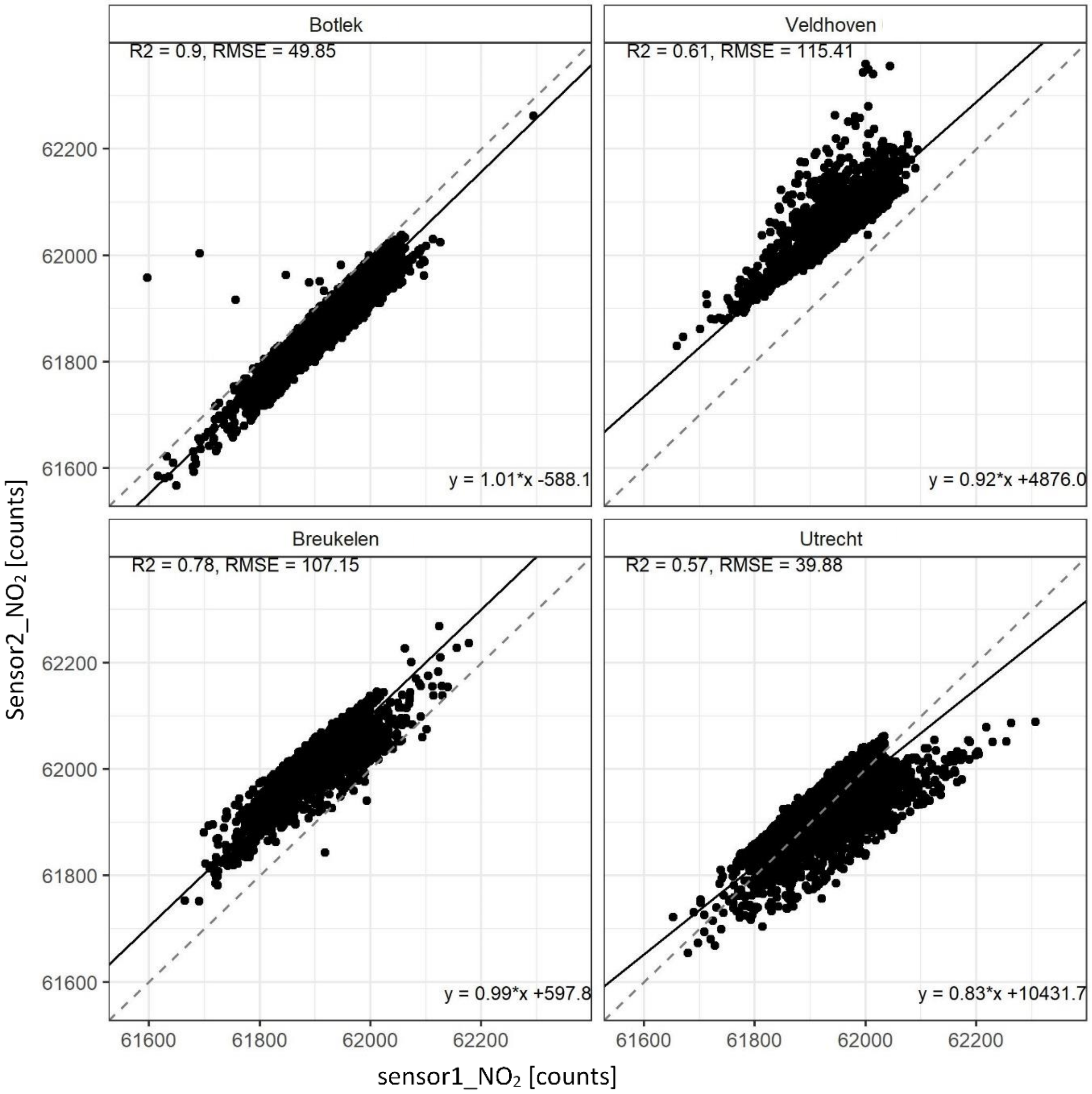

Figure S4. Scatter of NO_2 sensor signal of co-located sensor pairs at the four measurement locations.
